# Complete genome sequences of G*ordonia rubripertincta* phages OtterstedtS21 and Patos

**DOI:** 10.1128/MRA.00718-23

**Published:** 2023-09-29

**Authors:** Bowen Meng, Alexander Bleau, Riddhi R. Bombaywala, Audrey S. DeGraw, Manjot S. Deol, Kendall E. Dollard, Nicolas Gentile, Julianna Jebaraj, Gregory N. Kayayan, Briana C. Miranda, Ayobamidele E. Momoh, Elias Morales, Amalia C. Nunes, Antonia M. Oropallo, Sophia C. Otterstedt, Anna T. Pridell, Jenna I. Roberts, Gabriel A. Ruiz, Dhatri Sangasani, Renee D. Smith, Mahad Tarar, Vir Singh, Pradeepa Jayachandran

**Affiliations:** 1 Department of Basic and Clinical Sciences, Albany College of Pharmacy and Health Sciences, Albany, New York, USA; Portland State University, Portland, Oregon, USA

**Keywords:** bacteriophage, *Gordonia*

## Abstract

We report the genomes of two viruses with siphovirus morphology, OtterstedtS21 and Patos, from Albany, New York, using *Gordonia rubripertincta*. The genomes of OtterstedtS21 and Patos are ~68 kbp long with 58% GC content. Both phages group with cluster DV based on gene content similarity to phages in the Actinobacteriophage database.

## ANNOUNCEMENT

*Gordonia* are Gram-positive bacteria that belong to the actinobacterium phylum. Several members of *Gordonia* species have been observed to break down hydrocarbons and are thus potential bio-remediators that may be capable of reducing environmental pollution (1). Bacteriophages have also played incredibly important roles in shaping our understanding of microorganisms (2). To further our understanding of the genetic diversity and evolution of *Gordonia* phages, we report the discovery of two actinobacteriophages, OtterstedtS21 and Patos, which infect *G. rubripertincta*.

**Fig 1:**
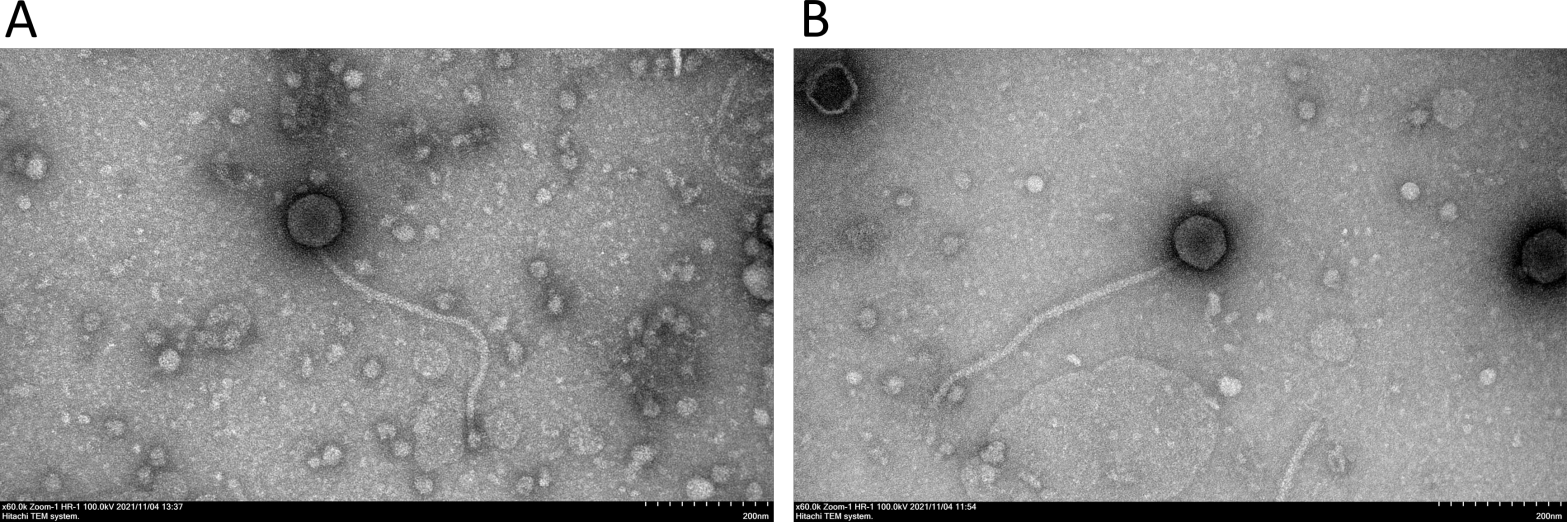
Electron micrographs of *Gordonia* phages OtterstedtS21 (**A**) and Patos (**B**). Negative stain (uranyl acetate, 1%) transmission electron microscopy of phages OtterstedtS21 (**A**) and Patos (**B**) reveals typical siphovirus morphology. The scale at bottom right of each image is 200 nm.

In the fall of 2021, phage OtterstedtS21 was isolated from soil collected from an active construction site near the Albany College of Pharmacy and Health Sciences Campus (42.646943°N, 73.778489°W) and phage Patos from soil collected at Lincoln Park in Albany, New York (42.644879°N, 73.76418°W), using standard procedures (2). In brief, each soil sample was mixed with PYCa (peptone, yeast extract, and calcium) liquid medium, filtered using a 0.22-µm filter, and the filtrate inoculated with *G. rubripertincta* and incubated with shaking at 30°C. After 2 days, the culture was centrifuged, and the supernatant was screened for phage by plating in PYCa top agar with *G. rubripertincta*. Both phages were purified through several rounds of plating (2). Transmission electron microscopy revealed both the phages to have siphovirus morphology (Fig. 1; Table 1).

**TABLE 1:** Characteristics of cluster DV siphoviruses OtterstedtS21 and Patos

Phage name	Number of reads (150 bp single base reads)	Coverage	Genome length	GC%	Capsid size	Tail length
OtterstedtS21	406,310	752×	67,688 bp	58.2%	~93 nm (*n* = 1)	~650 nm (*n* = 1)
Patos	503,759	4358×	68,847 bp	58.3%	~86 nm (*n* = 1)	~586 nm (*n* = 1)

DNA was extracted from OtterstedtS21 and Patos lysates using Wizard DNA Clean-Up Kit (Promega), prepared as sequencing libraries at the University of Pittsburgh Sequencing facility using the NEB Ultra-II Library Kit (New England Biolabs), and sequenced using an Illumina MiSeq (v3 reagents) to generate 150 base single-end reads as shown in Table 1. Raw reads were then assembled using Newbler v2.9 and verified for accuracy using Consed v29 (3). Both phages have circularly permuted genomes that share 95.2% nucleotide identity with each other (Table 1).

Autoannotation of the genome was performed using DNA Master v5.23.6 (4), Glimmer v 3.02b (5), and GeneMark v2.5p (6). Manual refinement of the gene calls was conducted using a combination of DNA Master v5.23.6 (4), Phamerator (7), and Starterator (http://phages.wustl.edu/starterator/). NCBI BLASTp (8) and HHPred (9) were used to assign gene functions, and TMHMM (10) and SOSUI (11) were used to identify potential membrane proteins (12). ARAGORN (13) and tRNAscan-SE (14) were used to identify potential tRNAs. All programs were used with default settings.

OtterstedtS21 and Patos are predicted to encode 98 and 102 protein-coding genes, respectively, all on the same strand, with no predicted tRNAs. Both phages share 91% gene content similarity (GCS) and, based on GCS of >35% to phages in the Actinobacteriophage database (phagesDB.org), both phages are grouped into bacteriophage cluster DV (15, 16). One half of both genomes encode virus structure and assembly functions, whereas the other half encodes for functions involved in DNA metabolism. Patos encodes for a DNA binding protein (gp64) and an HNH endonuclease (gp83) that are not present in OtterstedtS21. No immunity repressor or integrase functions could be identified in either phage, consistent with other cluster DV phages which suggest that neither phage is likely to establish lysogeny.

## Data Availability

OtterstedtS21 is available at GenBank with Accession No. OP172870 and Sequence Read Archive (SRA) No. SRX14483247. Patos is available at GenBank with Accession No. OP172876 and SRA No. SRX14483245.
